# Optimising irrigation strategies to improve soil water-salt distribution characteristics and enhance the cotton emergence rate of “dry sowing and wet emergence”

**DOI:** 10.3389/fpls.2026.1763080

**Published:** 2026-02-26

**Authors:** Xun Zhang, Fengquan Wu, Hao Zhang, Dong Wang, Yiming Zhang, Liangying Liu, Bingli Wang, Qiuxiang Tang

**Affiliations:** 1 Engineering Research Centre of Cotton, Ministry of Education/College of Agriculture, Xinjiang Agricultural University, Urumqi, China; 2 Xinjiang Qiangnong Fenghe Agricultural Technology Co., Ltd., Urumqi, Xinjiang, China

**Keywords:** dry sowing and wet emergence, irrigation frequency, irrigation volume, seedling emergence rate, soil salinity, soil water content

## Abstract

**Introduction:**

In arid regions with scarce water resources, water shortage constrains cotton production. “Dry sowing and wet emergence,” as a water-saving technique, promotes seed emergence by locally moistening the soil, but its application in saline-alkali areas is limited by unclear water-salt distribution patterns. This study hypothesized that optimized irrigation strategies could improve soil water-salt distribution and enhance emergence rates.

**Methods:**

To test this hypothesis, this study conducted a two-year (2024 and 2025) split-plot field experiment in the arid region of Xinjiang. The main plots were irrigation frequency: P1 (single drip irrigation on the first day after sowing) versus P2 (two drip irrigations on the first and eighth days after sowing); The subplots varied total irrigation volume: W1 (15 mm), W2 (30 mm), and W3 (45 mm). Irrigation was applied as a single application in P1 and divided equally into two applications in P2. The study examined the relationship between seedling-stage soil moisture content, salinity, uniformity of water-salt distribution, desalination rate, and emergence outcomes.

**Results:**

Two years of research indicate: Under identical irrigation frequencies, the W3 treatment increased soil moisture content in the 0–20 cm soil layer by 5.43% and 3.78% compared to W1 and W2, respectively. Water distribution uniformity improved, while salt content decreased by 4.76% and 11.28%, resulting in corresponding desalination rate increases of 25.02% and 7.67%. Under identical irrigation volumes, the P2 treatment exhibited a 7.42% higher soil moisture content in the 0–20 cm layer than the P1 treatment at 12 days post-sowing, with a 3.45% reduction in salinity and a 14.39% increase in emergence rate. Under synergistic regulation, the P2W2 treatment demonstrated the most optimal comprehensive performance. Compared to other treatments, it improved water uniformity by 1.05–6.28%, increased desalination rates by 15.62–32.27%, and boosted emergence rates by 5.79–13.91%. Correlation analysis revealed a significant positive correlation between emergence rate and moisture uniformity (R^2^ = 0.83) and a significant negative correlation with salinity uniformity (R²=0.82). Based on these relationships, this study established critical water-salt thresholds for ensuring emergence: maintaining soil moisture content between 18.36% and 19.82% in the 0–20 cm soil layer and keeping soil salinity below 3.65 g/kg ensures a stable emergence rate above 85%.

**Discussion:**

In summary, optimizing irrigation strategies (applying 15 mm of water on both the first and eighth days after sowing) improved soil water-salt distribution in the 0–20 cm soil layer by enhancing water retention capacity and promoting salt leaching, thereby increasing cotton emergence rates. This study provides theoretical and practical foundations for water-saving irrigation and water-salt regulation in cotton fields in southern Xinjiang.

## 1 Introduction

Cotton is a vital economic and fiber crop in China. In 2024, Xinjiang’s cotton output reached 5.686 million tons, accounting for 92.2% of the national total, solidifying its position as China’s premier cotton-producing region (Feng et al., 2024). Soil salinization remains a primary constraint on Xinjiang’s cotton industry development, particularly in the southern Xinjiang region (Liang et al., 2025). Traditional production practices widely employ extensive winter-spring flood irrigation to maintain soil moisture, leach soil salts, and ensure normal seed germination (Wang et al., 2025). However, stringent national controls on total agricultural water usage have significantly restricted winter-spring flood irrigation, hindering cotton emergence and subsequent growth (Ma et al., 2024). Consequently, advancing water-saving irrigation technologies in southern Xinjiang has become an urgent priority (Cao et al., 2020). The “dry sowing and wet emergence” technique, as an innovative water-saving irrigation method, precisely regulates soil moisture in the root zone through drip irrigation after sowing while enhancing soil water retention capacity and salt leaching efficiency. It has achieved remarkable results in northern Xinjiang’s cotton-growing regions. Relevant studies indicate this technology can stabilize seedling emergence rates above 85% and achieve average water savings of 30%-40%, the area under cultivation exceeds 7 million hectares, yet in the southern Xinjiang region it is only 1 million hectares (Ding et al., 2024; Jia et al., 2024). The climate and soil conditions in southern Xinjiang differ significantly from those in northern Xinjiang. This region experiences a lack of snow cover during winter, resulting in generally poor soil moisture conditions. The average moisture content in the 0–20 cm soil layer prior to sowing often falls below 12%, while soil salinity levels typically exceed 4.5 g/kg (Li et al., 2022). For cotton seedlings, salt stress affecting emergence and early growth occurs when surface soil salinity exceeds 4.0 g/kg (Yu et al., 2025).This imposes stricter threshold constraints on both irrigation volume and frequency for post-emergence irrigation, severely hindering the adoption of this technology (Dai et al., 2024; Jia et al., 2025).Therefore, conducting targeted research on optimizing irrigation regimes tailored to the unique water-salt conditions in southern Xinjiang, and identifying the water-salt thresholds and management practices necessary to ensure seedling emergence, holds urgent practical significance for advancing the adaptive promotion of this technology in the region.

Irrigation strategies are pivotal in regulating soil water-salt distribution characteristics, decisively influencing the migration and distribution of soil water and salts (He et al., 2025). Traditional flood irrigation readily induces deep percolation, leading to water resource wastage, while simultaneously causing groundwater level rise and secondary soil salinization (Hu et al., 2019; Li, 2020). Compared to flood irrigation, “dry sowing and wet emergence” achieves dual benefits of water conservation and salt control by precisely directing water supply to regulate the soil water-salt environment in the root zone, thereby preventing salt accumulation (Liang et al., 2025). However, insufficient irrigation volumes may result in inadequate water infiltration, leading to soil salt accumulation in the root zone and inducing salt stress; while excessive irrigation may drive upward migration of deep-soil salts and reduce root-zone soil aeration, impairing salt ion transformation and thereby increasing the risk of secondary salinization (Wang et al., 2025; Su et al., 2022). Furthermore, irrigation frequency significantly influences the spatial characteristics of water and salt distribution. Low-frequency irrigation promotes salt downward migration through enhanced gravitational leaching, temporarily improving surface soil salinity (Li et al., 2024; Yu et al., 2025). However, during irrigation intervals, transpiration triggers upward salt migration, causing accumulation in surface soils and ultimately forming ‘salt patches’ that impair cotton seedling emergence (Ma et al., 2025; Liang and Shi, 2021). Different irrigation strategies also directly or indirectly affect crop emergence rates. Appropriate irrigation practices create suitable water-salt conditions for seed germination, thereby improving emergence rates and uniformity. Dry sowing and wet emergence technology, through timely and appropriate irrigation after sowing, prevents seed rot caused by excessively wet soil during planting while ensuring seeds receive necessary moisture during the critical germination period. It also suppresses surface salinity accumulation, significantly improving crop emergence rates (Liang et al., 2025). Conversely, improper irrigation management severely inhibits emergence. Although infrequent irrigation promotes short-term salt leaching post-irrigation, prolonged intervals accelerate soil moisture evaporation. This causes salts to migrate upward with water and accumulate in the shallow soil layer where seeds reside, readily forming salt crusts or salt spots (He et al., 2023). These directly impede seed swelling, radicle elongation, and seedling emergence through the soil, resulting in delayed emergence, reduced emergence rates, and even gaps in rows (Nabi et al., 2001). Therefore, optimizing irrigation strategies is not only central to regulating soil water and salt distribution but also a critical agronomic measure for ensuring high-quality crop emergence and achieving robust, uniform seedlings.

Although existing research has separately elucidated the effects of irrigation volume and irrigation frequency on soil water-salt distribution and seedling emergence rates, a systematic quantitative study remains lacking on how these two factors synergistically regulate each other—particularly under dry-seeded, wet-emerged conditions in arid, saline-affected cotton-growing regions of southern Xinjiang—and on their optimal combination. Elucidating the coupled relationship between irrigation frequency and volume, along with their joint mechanism affecting emergence rates, holds significant implications for optimising water-saving and salt-control irrigation regimes and achieving stable high yields. This represents a critical research gap demanding urgent attention. Consequently, this study proposes the hypothesis that under dry-seeded, wet-emerged conditions, optimised irrigation strategies can improve soil water-salt distribution characteristics, thereby promoting cotton seedling emergence. To test this hypothesis, a two-year field trial was conducted in the southern cotton-growing region of Xinjiang. The primary objectives were: (a) to evaluate the regulatory effects of varying irrigation volumes and frequencies on soil water-salt distribution characteristics; (b) elucidate how varying irrigation volumes and frequencies influence water-salt distribution uniformity and desalination efficiency; (c) reveal the correlation between water-salt distribution characteristics and cotton seedling emergence outcomes, thereby providing theoretical foundations and technical support for enhancing water resource efficiency in southern Xinjiang’s dry sowing and wet emergence cotton cultivation practices.

## 2 Materials and methods

### 2.1 Overview of the pilot zone

**Figure 1:**
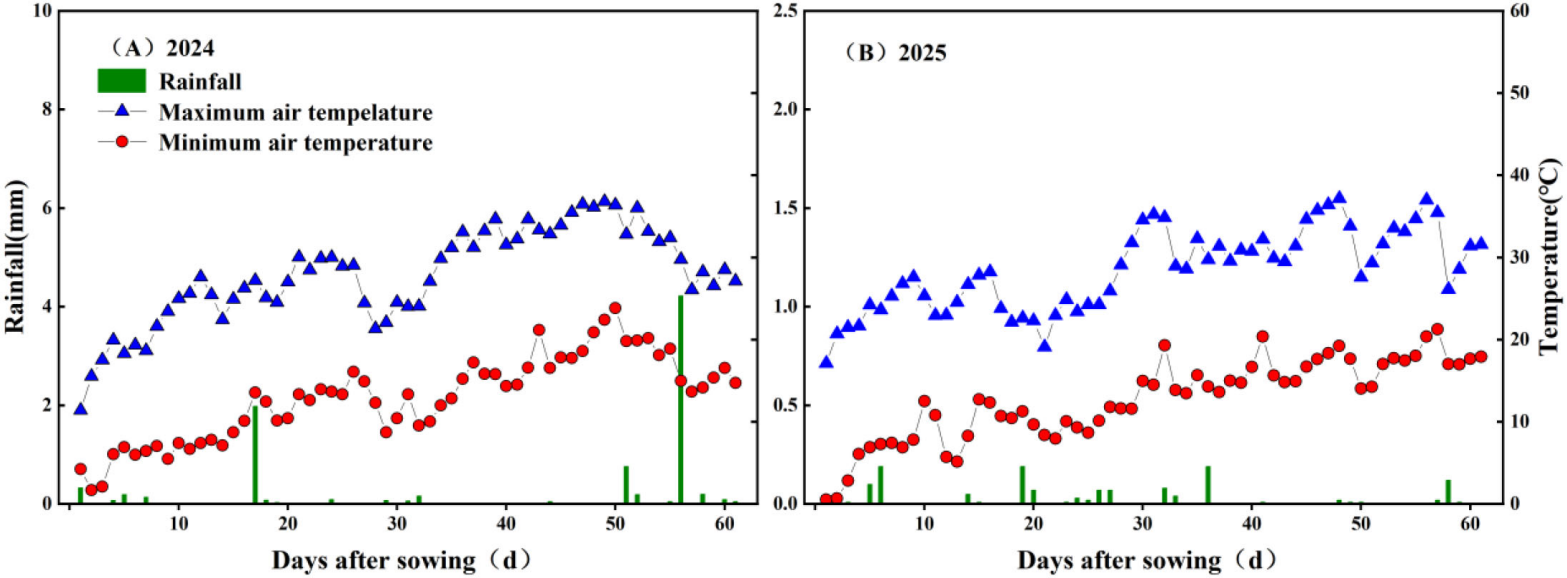
**(a)** Climate diagram showing precipitation, maximum temperature, and minimum temperature for the year 2024; **(b)** Same as **(a)** but for the year 2025.

The cotton variety used in this study is the locally predominant cultivar Xinluzhong 54. The experiment was conducted from 2024 to 2025 in Shaya County, Xinjiang (41°17′N, 82°42′E, elevation 897 meters). This region features a warm temperate continental arid climate, with an annual average precipitation of 47.3 mm, annual evaporation of 2000.7 mm, annual sunshine duration of 3031.2 hours, and an annual average temperature of 10.7 °C. The highest recorded temperature reached 30.9 °C, while the lowest dropped to -13.7 °C. The frost-free period spans 214 days. Daily minimum/maximum temperatures and precipitation during the study period are shown in **Figure 1**. The experimental site consists of sandy loam soil. In 2024, the soil’s initial moisture content was 16.13%, salt content was 3.98 g/kg, total nitrogen content was 0.65 g/kg, bulk density was 1.51 g/cm³, and pH was 8.26. In 2025, the initial moisture content was 14.38%, salt content was 4.16 g/kg, total nitrogen content was 0.63 g/kg, bulk density was 1.54 g/cm³, and pH was 8.45.

### 2.2 Experimental design

**Figure 2:**
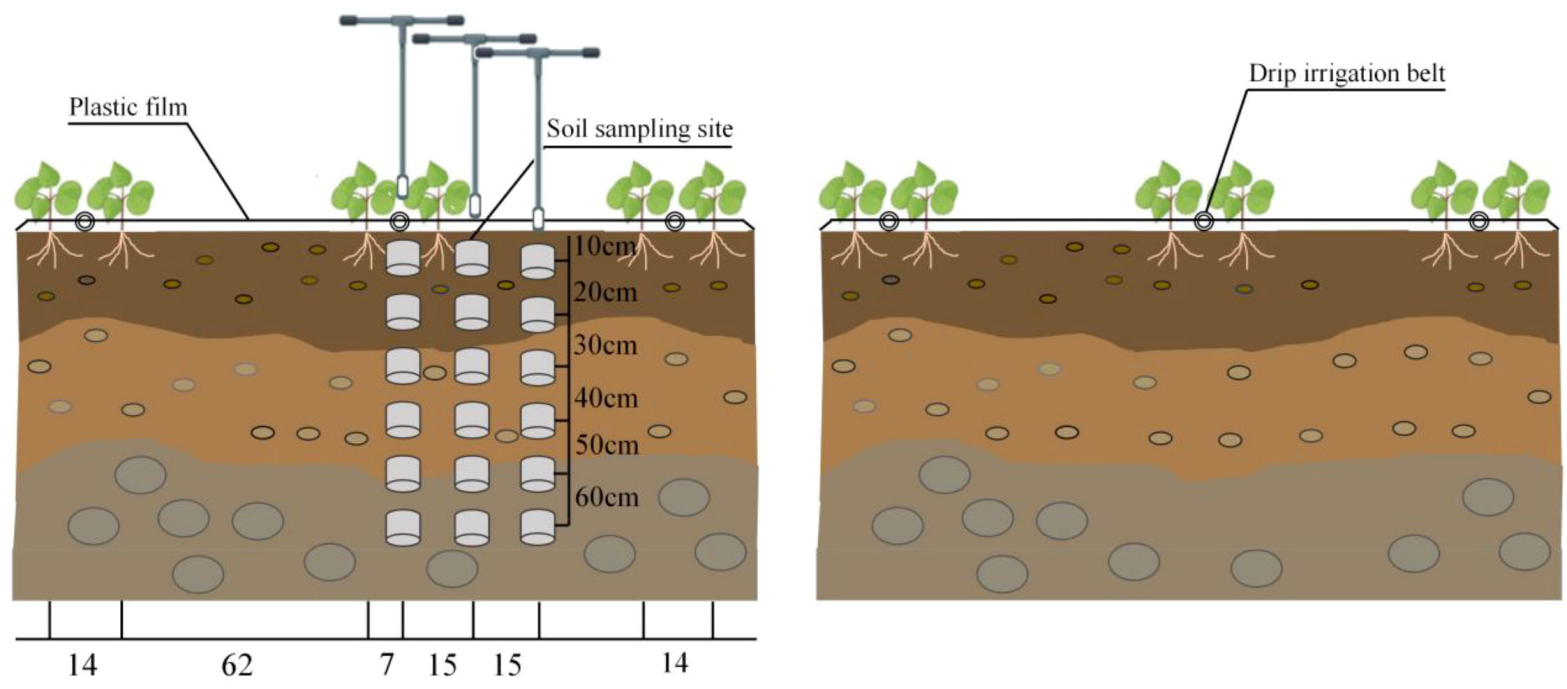
Planting pattern and sampling diagram.

This study employed a split-plot design, with the main plots assigned two irrigation frequencies (P1: Day 1 after sowing; P2: Day 1 + Day 8 after sowing) and the subplots assigned three irrigation volumes (W1: 15 mm, W2: 30 mm, W3: 45 mm). Irrigation was completed in a single application for P1 and divided equally into two applications for P2. The widely adopted production practice of a single 30 mm irrigation (P1W2) served as the control treatment. A total of six treatments were established, each replicated three times. Cotton was cultivated using a 1-film, 3-pipe, 6-row planting pattern. The plastic mulch width was 2.05 meters, with alternating row spacing (64 cm + 12 cm). Patch-type drip tape was laid in the narrow rows at a flow rate of 2.1 L/h. Each experimental plot covered three film widths, measuring 6.67 m × 10 m. To prevent water seepage between adjacent treatment zones, a 1-meter buffer zone was established between each treatment. Sowing dates were April 7, 2024, and April 9, 2025. Irrigation water sourced from local canal water had a mineralization degree of 0.79 g·L^-1^ in 2024 and 0.83 g·L^-1^ in 2025. Irrigation was conducted within one day after sowing. Detailed irrigation protocols are provided in **Table 1**, the plot layout diagram and sampling locations in **Figure 2**.

**Table 1:** Irrigation plans for 2024 and 2025.

Treatment	Irrigation frequency	Irrigation amount/(mm)	Irrigation timing	Total irrigation(mm)
P1W1	1	15 + 0	DAS1	15
P1W2	1	30 + 0	DAS1	30
P1W3	1	45 + 0	DAS1	45
P2W1	2	7.5 + 7.5	DAS1+DAS8	15
P2W2	2	15 + 15	DAS1+DAS8	30
P2W3	2	22.5 + 22.5	DAS1+DAS8	45

### 2.3 Test items and methods

#### 2.3.1 Emergence rate

At 7 and 14 days after cotton sowing, randomly select plots measuring 2.92 m × 2.28 m (length × width) to determine cotton emergence rates.


Emergencerate=Number of seedlings emerged/Total number of holes×100%

#### 2.3.2 Soil water content

Soil samples were collected using a 10 cm diameter soil auger to measure soil moisture. Sampling was conducted at 10 cm intervals (0–60 cm) at the following locations: directly beneath the drip emitters, 15 cm from the drip tape, and 30 cm from the drip tape. Soil sampling occurred on days 2, 4, 6, 8, 10, 12, 14, and 16 post-sowing. Fresh soil samples were weighed post-collection and dried in a fan-assisted oven at 105 ± 2 ^°^C to constant weight. Soil moisture content was calculated as follows (Xiao et al., 2023):


SWC(%)=(FW−DW)/DW×100

Where FW and DW denote the fresh weight and dry weight (g) of the soil sample respectively.

#### 2.3.3 Soil salt content

Soil salinity measurements were synchronised with soil moisture measurements in both timing and location. Following collection, soil samples were air-dried and sieved through a 2.0 mm mesh. Soil extract solutions were prepared at a 1:5 soil-to-water ratio, with electrical conductivity (EC) determined using a conductivity meter (Shanghai Yidian Technology Instrument Co., Ltd.). Corresponding salinity levels in the extract solutions were determined via the drying method. The formula is as follows (Isayenkov and Maathuis, 2019):


$$\text{S}=0.0061\times \text{EC}({\text{R}}^{2}=0.97)$$

Where: S denotes soil salinity content, g·kg^-1^; EC denotes electrical conductivity value, μs·cm^-1^.

#### 2.3.4 Soil desalination rate

The formula for calculating the desalination rate of soil salinity is as follows (Wei et al., 2022):


$$\text{Rs}=({\text{S}}_{1}-{\text{S}}_{2})/{\text{S}}_{1}\times 100\%$$

Where: S_1_ is the soil salinity content of the cotton field prior to irrigation, g·kg^-1^; S_2_ is the soil salinity content after irrigation, g·kg^-1^.

#### 2.3.5 Calculation of water and salt distribution

To assess the spatial uniformity of soil moisture content and soil salinity distribution, this study employs the Christiansen uniformity coefficient (*Cu)* as a quantitative indicator. This coefficient is determined through systematic spatial sampling of field soil properties, based on the following principle: By calculating the arithmetic mean of all observed values from sampling points and the mean absolute deviation of each observation relative to this mean, the spatial dispersion of soil indicators relative to their average state can be quantified. This enables assessment of the uniformity in soil moisture and salinity distribution. The formula for calculating the Christiansen uniformity coefficient (*Cu*) is as follows (Green and Pattison, 2022):


$$Cu=1-\frac{\sum_{i=1}^{n}|X_{i}-\bar{x}|}{n\bar{x}}$$

Where: *Cu* denotes the Christiansen uniformity coefficient, %; *Xi* represents the *i*-th soil observation value, g·kg^-1^; $\bar{x}$ denotes the mean of soil observation values, g·kg^-1^; *n* denotes the number of tests.

### 2.4 Data processing

Data were organised and calculated using Excel 2023. Analysis of variance (ANOVA), Pearson correlation analysis, and linear fitting were performed using SPSS 2024. Plotting was conducted using Origin 2024 and Surfer 15.0.

## 3 Results and analysis

### 3.1 Soil moisture

**Figure 3:**
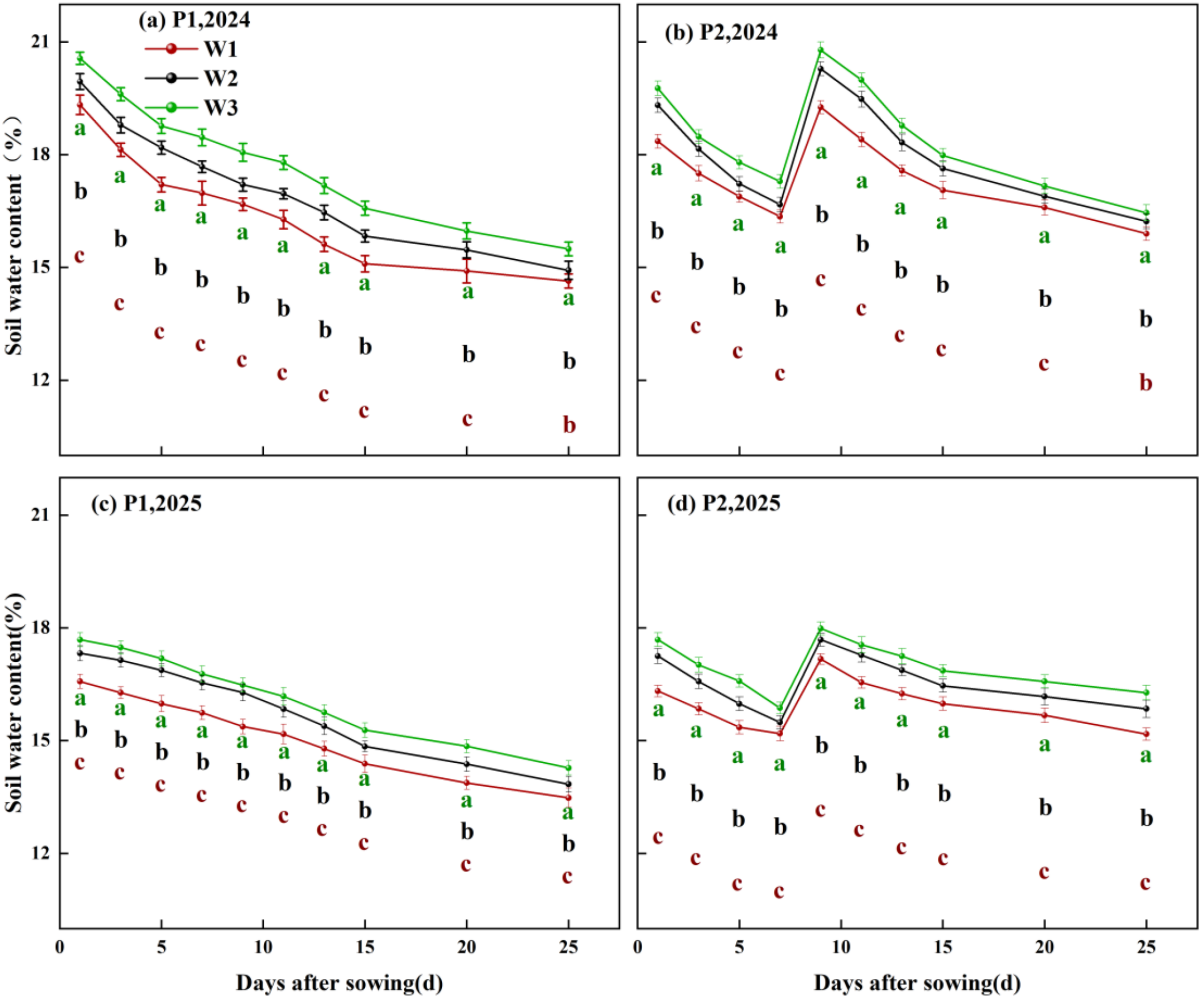
Time-dependent changes in average soil moisture content at the 0–60 cm depth under different irrigation frequencies and volumes. P1 and P2 denote single irrigation and double irrigation, respectively; W1, W2, and W3 represent irrigation volumes of 15 mm, 30 mm, and 45 mm, respectively; **(a–c)** indicate significant differences between treatments (p < 0.05), **(d)** denotes days after sowing.

Both irrigation frequency and irrigation volume exerted significant effects on soil moisture content in cotton fields (**Figure 3**, P<0.05). Findings indicate that soil moisture content in 2024 increased by 2.46% compared to 2025. Results from both years demonstrate that, under identical irrigation volumes, increasing irrigation frequency significantly elevates average soil moisture content. Compared with the P1 treatment, the P2 treatment increased soil moisture content by 6.22% to 8.62%. At the same irrigation frequency, average soil moisture content showed an upward trend with increasing irrigation volume. Under P1 conditions, the average soil moisture content of the W3 treatment increased by 5.83% and 3.79% compared with W2 and W1, respectively. Under P2 conditions, the W3 treatment exhibited soil moisture content 2.68% and 3.49% higher than W2 and W1 respectively. Under the synergistic regulation of irrigation frequency and volume, the P2W3 treatment achieved the highest average soil moisture content over two years, exceeding other treatments by 3.28% to 12.37%.

**Figure 4:**
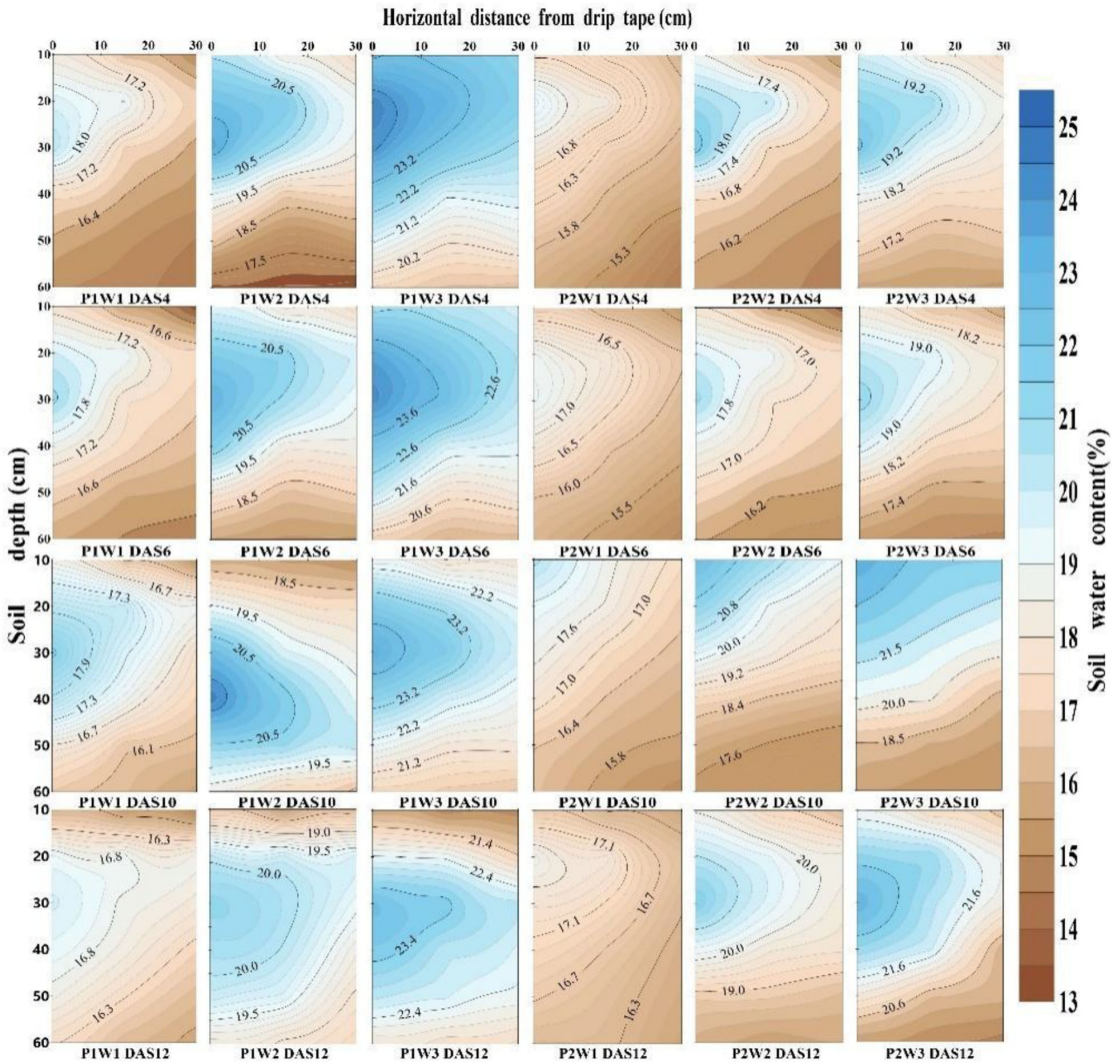
Soil moisture distribution in the 0–60 cm soil profile under different irrigation frequencies and volumes in 2024. P1 and P2 denote single and double irrigation, respectively; W1, W2, and W3 represent irrigation volumes of 15 mm, 30 mm, and 45 mm, respectively; DAS 4, 6, 10, 12 denote 4 days, 6 days, 10 days, and 12 days after sowing respectively.

**Figure 5:**
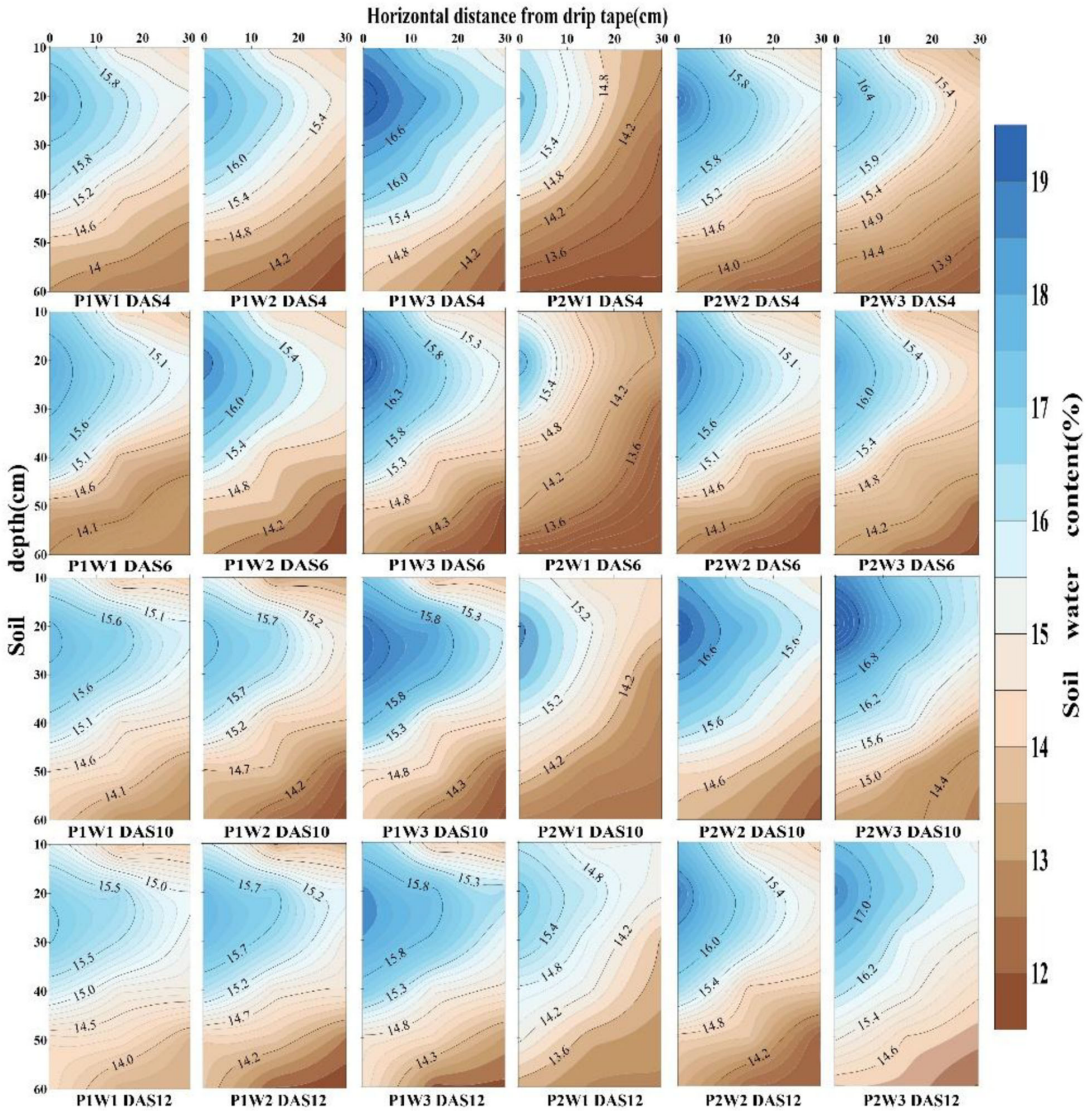
Soil moisture distribution in the 0–60 cm soil profile under different irrigation frequencies and volumes in 2025. P1 and P2 denote single and double irrigation, respectively; W1, W2, and W3 represent irrigation volumes of 15 mm, 30 mm, and 45 mm, respectively; DAS 4, 6, 10, 12 denote 4 days, 6 days, 10 days, and 12 days after sowing respectively.

Further analysis of the spatiotemporal distribution of soil moisture revealed (**Figures 4**, **5**) that irrigation volume and frequency significantly influenced soil moisture in the 0–20 cm soil layer. Vertically, soil moisture content in the 0–20 cm layer was markedly higher than in the 20–60 cm layer. On days 6 and 12 post-sowing, the W3 treatment exhibited average increases in soil moisture content within the 0–20 cm layer of 15.26% and 35.38% compared to W2, and 12.99% and 32.08% compared to W1, respectively. Horizontally, soil moisture content at 0 cm from the dripper (narrow row spacing) was significantly higher than at 30 cm (wide row spacing), with this difference gradually diminishing as irrigation volume and frequency increased. Specifically, on days 6 and 12 post-sowing, under identical irrigation frequency, soil moisture content at 30 cm horizontally in the W3 treatment was on average 14.72% and 32.87% higher than in W2 and W1 respectively. Under the combined regulation of irrigation frequency and volume, at 6 days post-sowing, the soil moisture content at 30 cm horizontally and 20 cm vertically from the dripper in the P2W3 treatment was 21.93% lower than that in P1W3. However, at 12 days post-sowing, the soil moisture content in the P2W3 treatment was 6.73% higher horizontally and 5.48% higher vertically than that in P1W3.

### 3.2 Uniformity of soil moisture distribution

The results over the past two years indicate: Both irrigation volume and irrigation frequency exerted significant effects on copper content in the 0–20 cm soil layer (**Table 2**, P<0.05). At identical irrigation volumes, increasing irrigation frequency markedly enhanced soil moisture uniformity at 10 and 12 days post-sowing. Specifically, under treatment P2, Cu levels in the 0–20 cm soil layer at 10 and 12 days post-sowing increased by 1.05%–3.26% and 1.04%–2.08%, respectively, compared to treatment P1. At constant irrigation frequency, increasing irrigation volume also enhanced moisture uniformity. Under P1 conditions, the Cu value in the 0–20 cm soil layer for the W3 treatment was 5.83% and 3.79% higher than that of W2 and W1, respectively. Under P2 conditions, the Cu value in soil moisture content for the W3 treatment was 2.68% and 3.49% higher than that of W2 and W1, respectively. When both factors were jointly regulated, the P2W3 treatment yielded the optimal results: at 10 and 12 days post-sowing, the Cu values in the 0–20 cm soil layer reached 0.95 and 0.98, respectively, representing increases of 3.49% to 12.41% compared to other treatments.

**Table 2:** Variations in the uniformity of soil moisture distribution within the 0–20 cm layer of cotton fields under different irrigation frequencies and volumes.

Year	Treatment		DAS4	DAS6	DAS10	DAS12
2024	P1	W1	0.85 ± 0.01c	0.88 ± 0.03b	0.91 ± 0.02b	0.95 ± 0.01b
		W2	0.87 ± 0.02b	0.89 ± 0.02b	0.92 ± 0.01b	0.96 ± 0.02ab
		W3	0.91 ± 0.02a	0.93 ± 0.02a	0.94 ± 0.01a	0.96 ± 0.01a
	P2	W1	0.77 ± 0.03d	0.83 ± 0.02c	0.93 ± 0.02ab	0.94 ± 0.02b
		W2	0.85 ± 0.01c	0.88 ± 0.01b	0.95 ± 0.02a	0.97 ± 0.02a
		W3	0.88 ± 0.02b	0.89 ± 0.02b	0.95 ± 0.02a	0.98 ± 0.03a
2025	P1	W1	0.79 ± 0.01c	0.81 ± 0.02c	0.85 ± 0.02c	0.88 ± 0.01b
		W2	0.82 ± 0.01b	0.85 ± 0.01b	0.88 ± 0.01ab	0.90 ± 0.02ab
		W3	0.85 ± 0.02a	0.87 ± 0.02a	0.89 ± 0.02a	0.91 ± 0.03a
	P2	W1	0.75 ± 0.01d	0.78 ± 0.01d	0.81 ± 0.02d	0.83 ± 0.02c
		W2	0.79 ± 0.02c	0.81 ± 0.02c	0.87 ± 0.01b	0.90 ± 0.02ab
		W3	0.83 ± 0.02ab	0.86 ± 0.01a	0.90 ± 0.02a	0.92 ± 0.01a
Y			**	**	**	**
W			**	*	*	*
D			*	*	*	*
W*D			**	*	*	*
Y*D*W			**	**	**	*

Different lowercase letters denote significant differences in soil moisture uniformity among treatments (P<0.05). DAS4: 4 days after sowing, DAS6: 6 days after sowing, DAS10: 10 days after sowing, DAS12: 12 days after sowing. Y denotes different years, D denotes irrigation frequency, W denotes irrigation volume. * denotes significant differences between treatments (P<0.05), ** denotes highly significant differences between treatments (P<0.01).

### 3.3 Soil salinity

**Figure 6:**
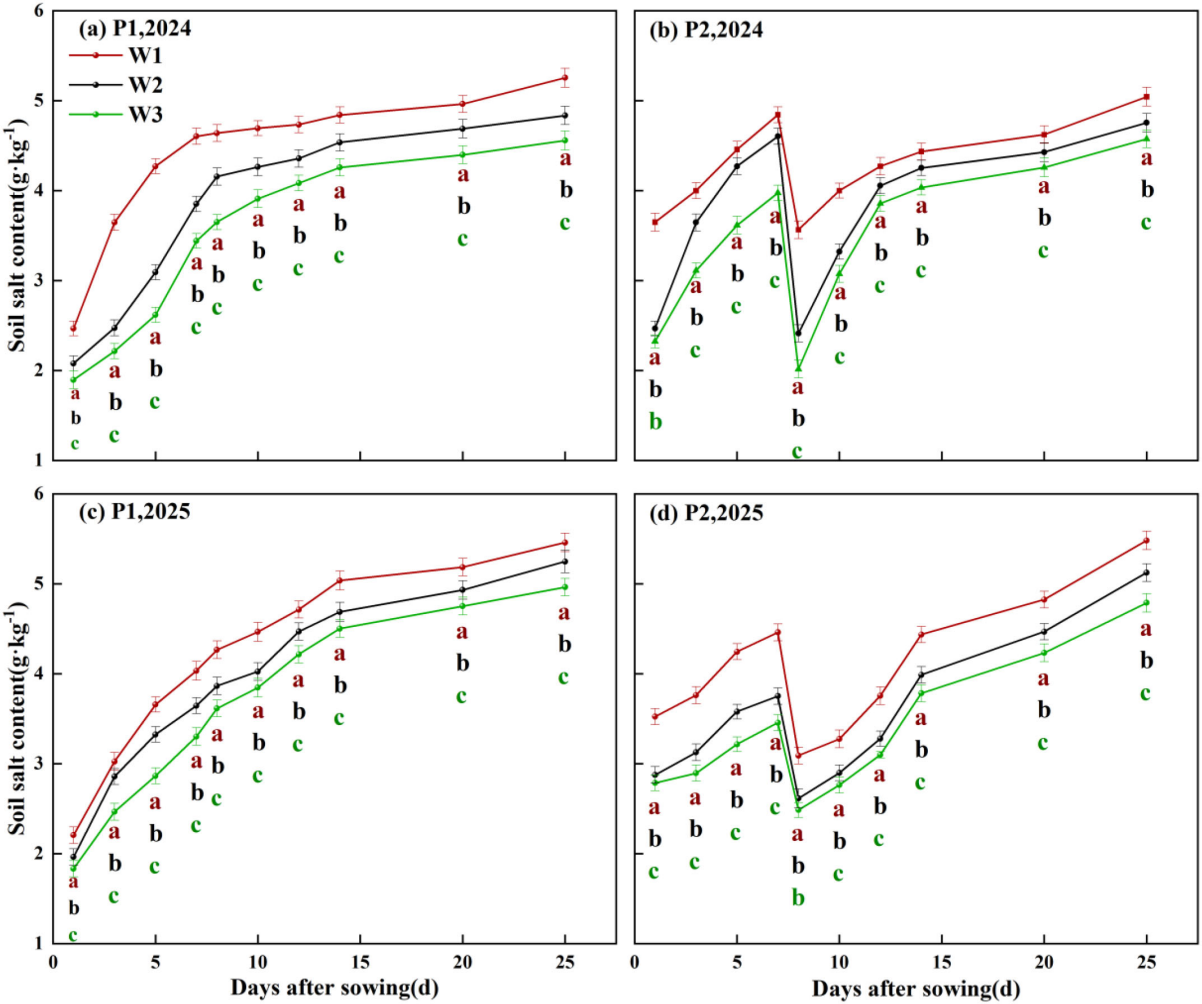
Time-dependent changes in average soil moisture content at the 0–60 cm soil layer under different irrigation frequencies and irrigation volumes. P1 and P2 denote single irrigation and double irrigation, respectively; W1, W2, and W3 represent irrigation volumes of 15 mm, 30 mm, and 45 mm, respectively; **(a–c)** indicate significant differences between treatments (p < 0.05), **(d)** denotes days after sowing.

Both irrigation frequency and irrigation volume exerted significant effects on soil salinisation in cotton fields (**Figure 6**, P<0.05). Findings indicate that soil salinity decreased by 5.81% in 2024 compared to 2025. Results from both years demonstrate that increasing irrigation frequency while maintaining constant irrigation volume significantly reduces average soil salinisation levels. Compared with the P1 treatment, soil salinity decreased by 1.66% to 5.23% under the P2 treatment. At the same irrigation frequency, soil salinity showed a decreasing trend with increasing irrigation volume. Under the P1 condition, the W3 treatment reduced soil salinity by 5.73% and 13.27% compared with the W2 and W1 treatments, respectively. Under P2 conditions, the W3 treatment exhibited soil salinity levels 3.78% and 9.29% lower than W2 and W1 respectively. Under the combined effects of irrigation volume and frequency, the P2W3 treatment recorded the lowest average soil salinity over two years, reducing levels by 3.78% to 12.94% compared to other treatments.

**Figure 7:**
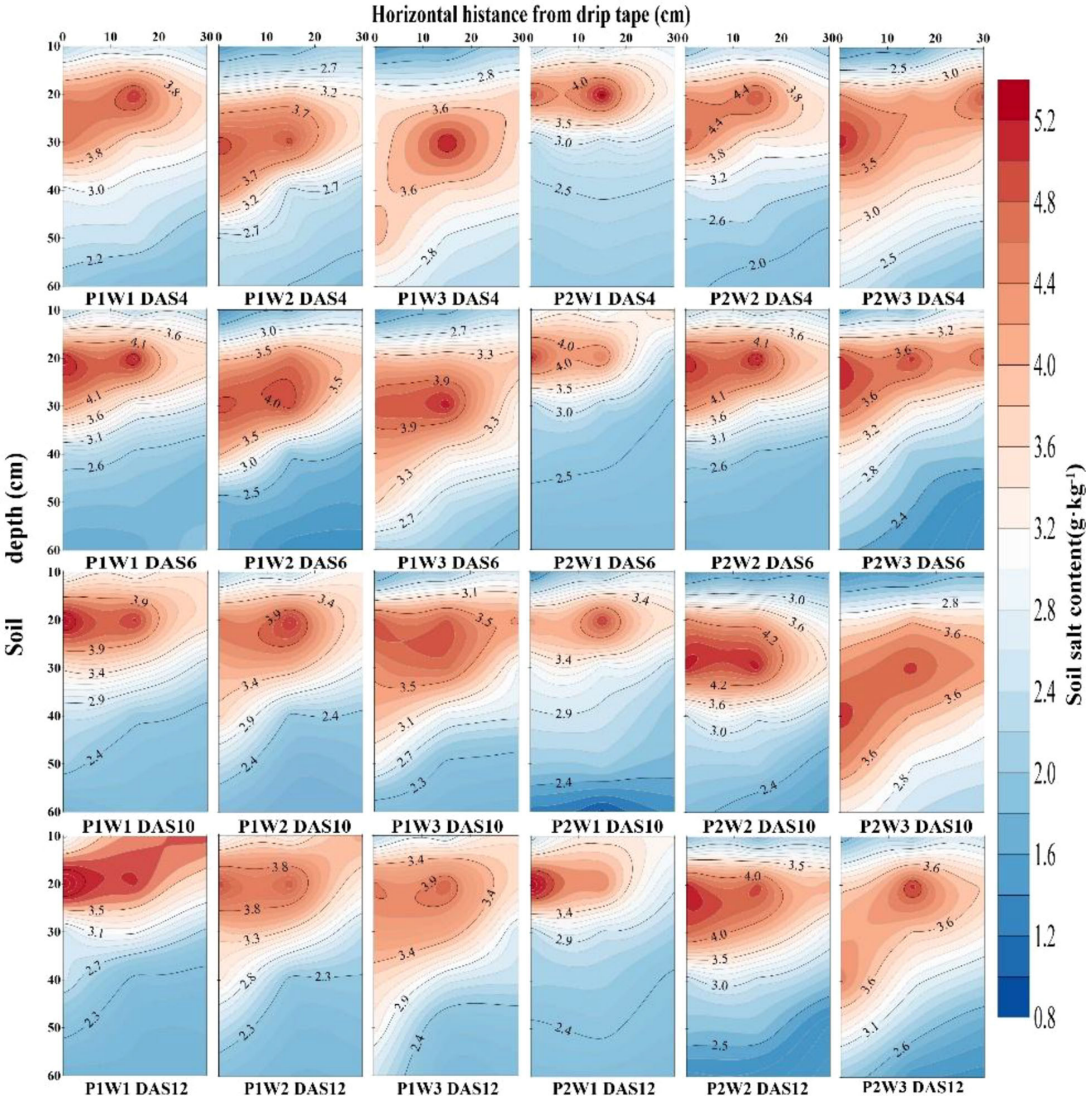
Soil salinity distribution in the 0–60 cm soil profile under different irrigation frequencies and volumes in 2024. P1 and P2 denote single and double irrigation, respectively; W1, W2, and W3 represent irrigation volumes of 15 mm, 30 mm, and 45 mm, respectively; DAS 4, 6, 10, 12 denote 4, 6, 10, and 12 days after sowing respectively.

**Figure 8:**
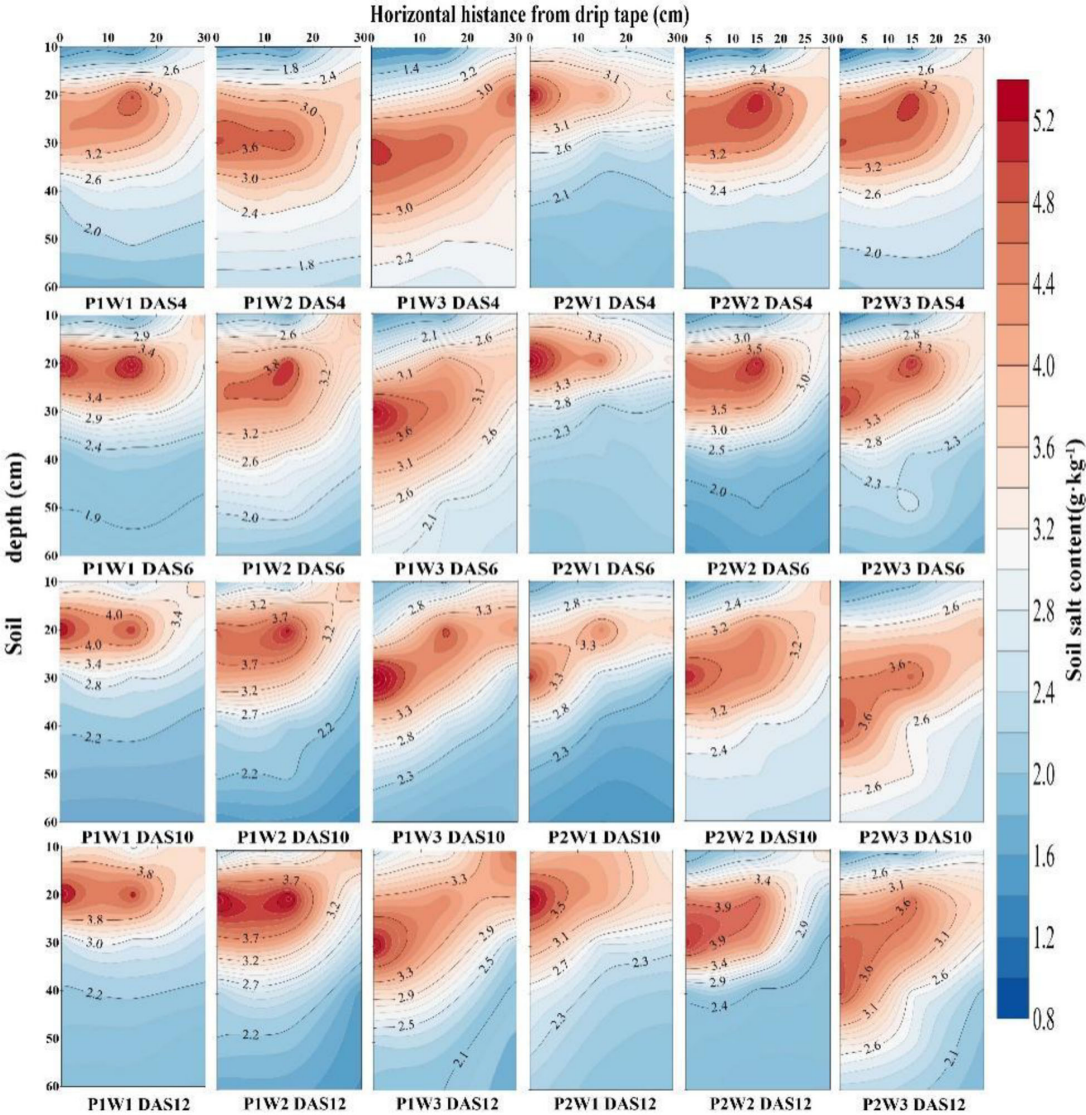
Soil salinity distribution in the 0–60 cm soil profile under different irrigation frequencies and volumes in 2025. P1 and P2 denote single and double irrigation, respectively; W1, W2, and W3 represent irrigation volumes of 15 mm, 30 mm, and 45 mm, respectively; DAS 4, 6, 10, 12 denote 4 days, 6 days, 10 days, and 12 days after sowing respectively.

Further analysis of the spatial distribution of soil salinity revealed (**Figures 7**, **8**) that irrigation volume and frequency significantly influenced soil salinity in the 0–20 cm soil layer. Vertically, salinity levels in the 0–20 cm layer were markedly lower than those in the 20–60 cm layer. On days 6 and 12 post-sowing, W3 exhibited average reductions in soil salinity within the 0–20 cm layer of 2.21% and 7.36% respectively compared to W2, and 3.27% and 4.19% compared to W1. Horizontally, soil salinity at 0 cm from the dripper (narrow row) was significantly lower than at 30 cm (wide row), with the difference gradually diminishing as irrigation volume and frequency increased. Specifically, on days 6 and 12 post-sowing, under identical irrigation frequency, soil salinity at 30 cm horizontally in W3 was on average 5.53% and 12.35% lower than in W2 and W1 respectively. Under the combined regulation of irrigation frequency and volume, at 6 days post-sowing, the soil salinity at 30 cm horizontally and 20 cm vertically from the dripper in the P2W3 treatment was 7.21% higher than that in P1W3. However, at 12 days post-sowing, the soil salinity in the P2W3 treatment was 11.64% and 9.56% lower than that in P1W3 in the horizontal and vertical directions, respectively.

### 3.4 Uniformity of soil salinity

Both irrigation volume and irrigation frequency significantly influenced soil salinity in the 0–20 cm soil layer (**Table 3**, P<0.05). At identical irrigation volumes, increasing irrigation frequency markedly reduced soil salinity uniformity in the 0–20 cm layer at 9 and 12 days post-sowing. Compared to the P1 treatment, the P2 treatment exhibited reductions in Cu content within the 0–20 cm soil layer of 8.69–16.66% and 6.59–14.28% at 10 and 12 days post-sowing, respectively. At the same irrigation frequency, increasing irrigation volume reduced soil salinity uniformity in the 0–20 cm layer. Under P1 conditions, the Cu value for soil moisture content in the 0–20 cm layer under treatment W3 decreased by 4.21% and 6.18% compared to W2 and W1, respectively. Under P2 conditions, the Cu content in the 0–20 cm soil layer of the W3 treatment was 2.29% and 1.16% lower than that of W2 and W1, respectively. Under the combined regulation of irrigation frequency and volume, the Cu value of soil salinity in the 0–20 cm layer under P2W3 reached 0.84 and 0.85 at 10 and 12 days post-sowing, respectively, representing reductions of 3.49% to 12.41% compared to other treatments.

**Table 3:** Variation in the uniformity of soil salinity distribution at 0–20 cm depth in cotton fields under different irrigation frequencies and volumes.

Year	Treatment		DAS4	DAS6	DAS10	DAS12
2024	P1	W1	0.89 ± 0.02a	0.93 ± 0.02a	0.96 ± 0.02a	0.97 ± 0.02a
		W2	0.81 ± 0.03b	0.87 ± 0.01b	0.94 ± 0.02b	0.95 ± 0.03b
		W3	0.80 ± 0.01c	0.86 ± 0.01b	0.92 ± 0.01c	0.91 ± 0.03c
	P2	W1	0.80 ± 0.02bc	0.84 ± 0.02c	0.80 ± 0.02e	0.87 ± 0.02d
		W2	0.89 ± 0.03a	0.93 ± 0.02a	0.85 ± 0.02d	0.86 ± 0.02de
		W3	0.88 ± 0.02a	0.92 ± 0.01a	0.84 ± 0.01d	0.85 ± 0.01e
2025	P1	W1	0.67 ± 0.02b	0.79 ± 0.02b	0.86 ± 0.01b	0.89 ± 0.02ab
		W2	0.60 ± 0.03e	0.70 ± 0.02d	0.83 ± 0.02c	0.85 ± 0.01c
		W3	0.62 ± 0.02d	0.71 ± 0.02d	0.83 ± 0.02c	0.88 ± 0.02b
	P2	W1	0.81 ± 0.03a	0.85 ± 0.01a	0.88 ± 0.02a	0.90 ± 0.01a
		W2	0.67 ± 0.02b	0.79 ± 0.02b	0.68 ± 0.02d	0.76 ± 0.02d
		W3	0.65 ± 0.02c	0.77 ± 0.01c	0.64 ± 0.03e	0.75 ± 0.02d
Y			**	**	**	**
W			**	*	*	*
D			**	*	*	*
W*D			**	*	*	*
Y*D*W			**	**	*	*

Different lowercase letters denote significant differences in soil salinity uniformity among treatments (P<0.05). DAS4: 4 days after sowing, DAS6: 6 days after sowing, DAS10: 10 days after sowing, DAS12: 12 days after sowing. Y denotes different years, D denotes irrigation frequency, W denotes irrigation volume. * denotes significant differences between treatments (P<0.05), ** denotes highly significant differences between treatments (P<0.01).

### 3.5 Soil salinity mitigation effect

Both irrigation volume and irrigation frequency significantly influenced the desalination effect on soil salinity in the 0–20 cm soil layer (**Table 4**, P<0.05). At identical irrigation volumes, the desalination effect in the 0–20 cm soil layer exhibited an upward trend with increasing irrigation frequency, with treatment P2 showing an improvement of 11.62% to 34.79% compared to treatment P1. At constant irrigation frequency, the desalination effect increased incrementally with rising irrigation volume. Under P1 conditions, the W3 treatment exhibited a 12.63% and 8.05% reduction in desalination effectiveness compared to W1 and W2, respectively. Under P2 conditions, the W3 treatment showed a 37.42% and 7.28% decrease relative to W1 and W2. Under the combined effects of irrigation volume and frequency, at DAS 12, the P2W2 treatment achieved a 48.06% reduction in salinity in the 0–20 cm soil layer. This represented a 6.78% improvement over the P2W3 treatment and a 20.17%–35.34% increase compared to other treatments.

**Table 4:** Variation in desalination rate (%) of the 0–20 cm soil layer in cotton fields under different irrigation frequencies and irrigation volumes.

Year	Treatment		DAS4	DAS6	DAS10	DAS12
2024	P1	W1	52.77 ± 0.33d	41.56 ± 0.24d	38.40 ± 0.13f	34.57 ± 0.23f
		W2	56.37 ± 0.18b	48.49 ± 0.23a	39.43 ± 0.18e	36.50 ± 0.18e
		W3	60.77 ± 0.08a	46.60 ± 0.18b	41.34 ± 0.11d	37.45 ± 0.16d
	P2	W1	44.61 ± 0.47e	38.31 ± 0.12e	46.51 ± 0.26c	38.59 ± 0.20c
		W2	52.52 ± 0.21d	41.49 ± 0.19d	56.33 ± 0.14b	49.20 ± 0.12a
		W3	54.55 ± 0.25c	44.40 ± 0.14c	57.32 ± 0.15a	50.66 ± 0.20a
2025	P1	W1	62.54 ± 0.18d	53.66 ± 0.10d	36.45 ± 0.17e	36.45 ± 0.17e
		W2	65.32 ± 0.18b	56.47 ± 0.19c	37.53 ± 0.24d	37.53 ± 0.24d
		W3	67.46 ± 0.13a	60.39 ± 0.13a	42.54 ± 0.22c	42.54 ± 0.22c
	P2	W1	44.53 ± 0.29e	31.57 ± 0.15f	36.45 ± 0.16e	36.45 ± 0.16e
		W2	62.36 ± 0.21d	52.47 ± 0.20e	51.40 ± 0.21b	51.40 ± 0.21a
		W3	64.60 ± 0.15c	57.85 ± 0.16b	52.46 ± 0.21a	52.46 ± 0.21a
Y			**	**	**	**
W			**	*	*	*
D			**	*	**	*
W*D			**	*	**	*
Y*D*W			**	**	**	*

Different lowercase letters indicate significant differences in soil salinity reduction rates among treatment levels (P<0.05). DAS4: 4 days after sowing, DAS6: 6 days after sowing, DAS10: 10 days after sowing, DAS12: 12 days after sowing. Y denotes different years, D denotes irrigation frequency, W denotes irrigation volume. * denotes significant differences between treatments (P<0.05), ** denotes highly significant differences between treatments (P<0.01).

### 3.6 Cotton seedling emergence rate

**Figure 9:**
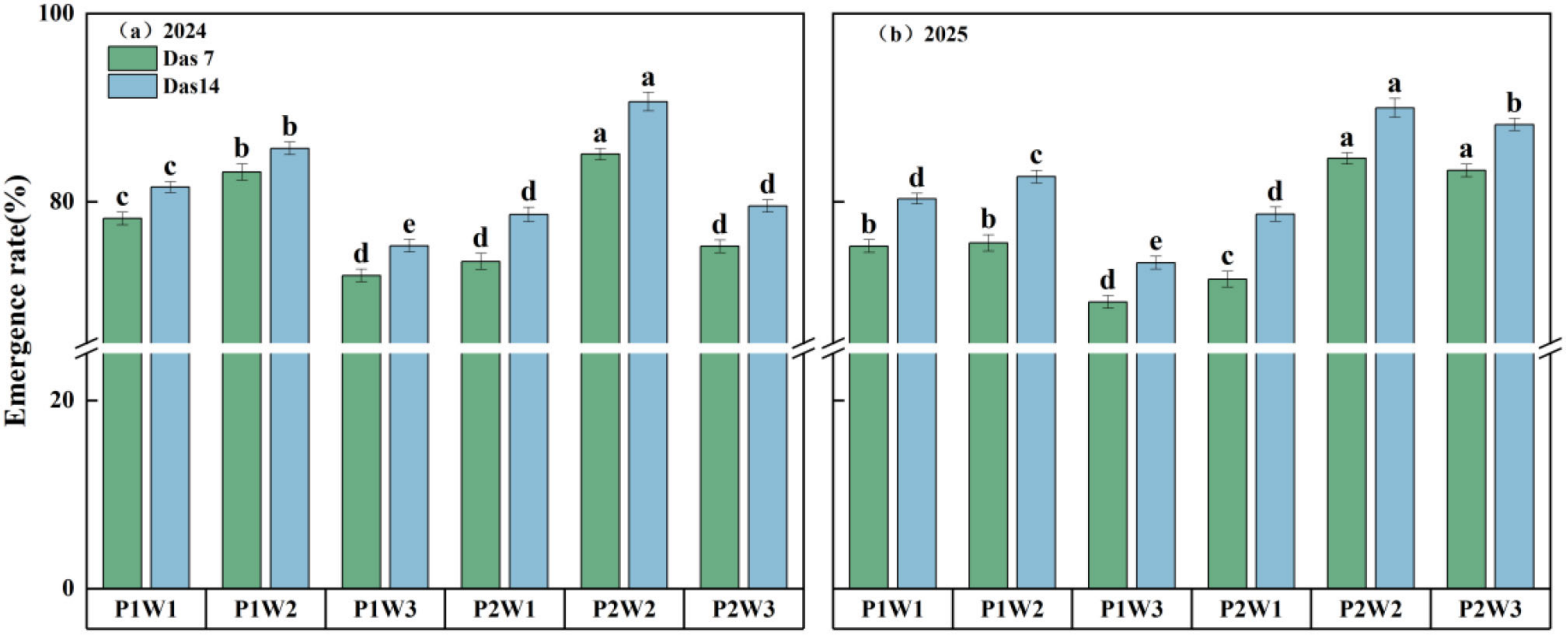
Cotton emergence rates under different irrigation frequencies and volumes in 2024 and 2025 P1 and P2 denote single irrigation and double irrigation respectively; W1, W2, and W3 represent irrigation volumes of 15 mm, 30 mm, and 45 mm respectively; DAS7: 7 days after sowing; DAS14: 14 days after sowing; (a–e) indicate significant differences between treatments (p < 0.05). **(a, b)** represent the cotton emergence rates for 2024 and 2025 respectively. Letters a, b, c, d, and e denote significant differences between treatment groups (p < 0.05).

Both irrigation volume and frequency significantly influenced cotton seedling emergence rates (**Figure 9**). Findings revealed that the average germination rate in 2024 exceeded that of 2025 by 1.59%. Results from both years indicate that, at constant irrigation volume, increasing irrigation frequency tends to enhance emergence rates. Compared to the P1 treatment, the P2 treatment showed emergence rate increases ranging from 8.84% to 19.95%. Data from both years demonstrate that, at constant irrigation frequency, cotton seedling emergence rates initially rise then decline with increasing irrigation volume. The W2 treatment exhibited a germination rate 2.91% higher than W1 and 12.41% higher than W3. Under the synergistic effect of irrigation volume and frequency, the P2W2 treatment achieved an average germination rate of 90.63% over two years, surpassing other treatments by 5.79% to 13.91%.

### 3.7 Correlation analysis between soil water-salt distribution characteristics and cotton seedling emergence rate

**Figure 10:**
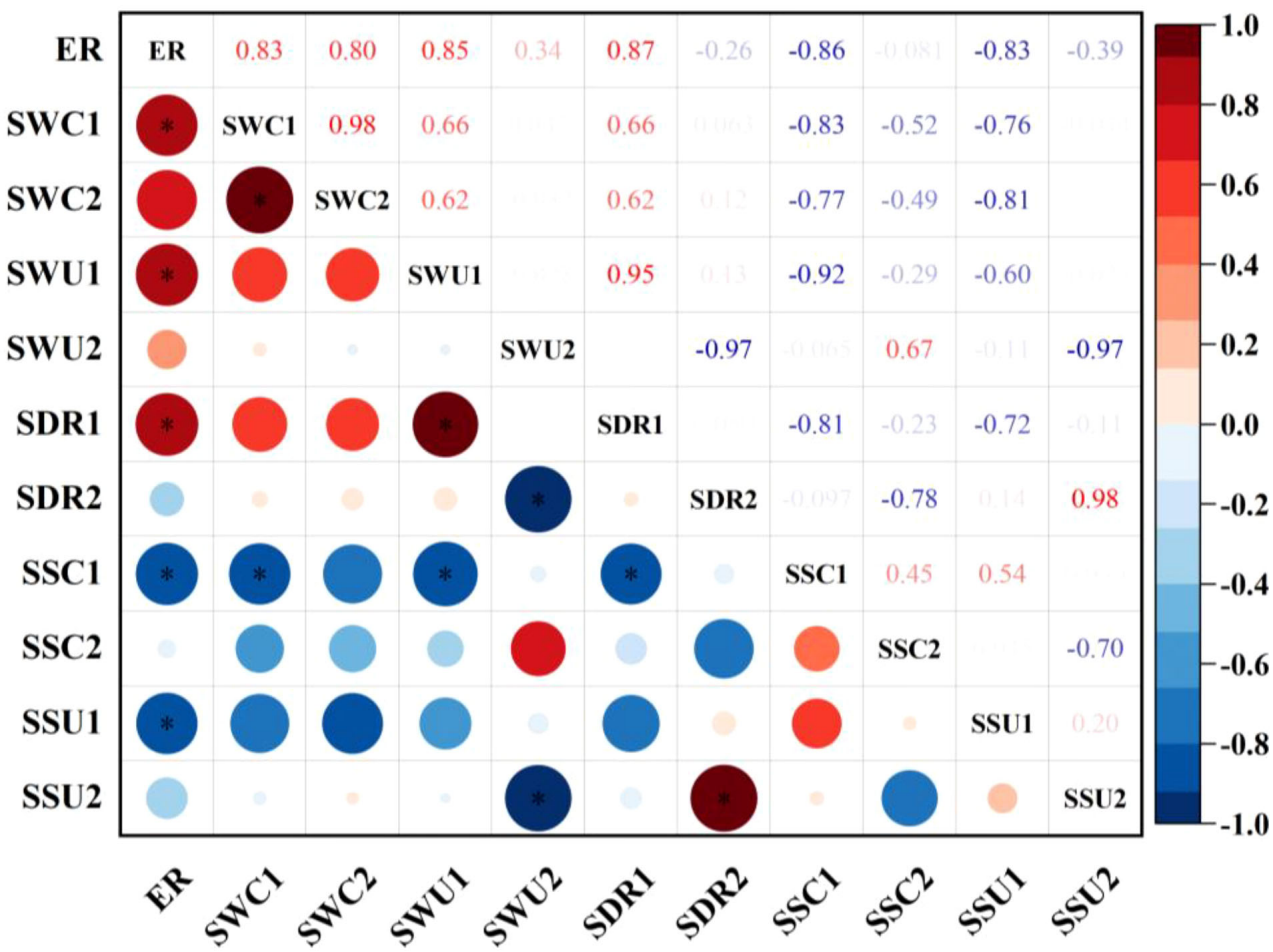
Correlation analysis between soil water-salt characteristics and seedling emergence rate. ER: Emergence rate, SWC1: Soil water content at 0–20 cm depth, SWC2: Soil water content at 20–40 cm depth, SWU1: Uniformity of water distribution in the 0–20 cm soil layer, SWU2: Uniformity of water distribution in the 20–40 cm soil layer, SDR1: Soil desalination rate at 0–20 cm depth, SDR2: Soil desalination rate at 20–40 cm depth, SSC1: Soil salinity content in the 0–20 cm layer, SSC2: Soil salinity content in the 20–40 cm layer, SSU1: Uniformity of salt distribution in the 0–20 cm soil layer, SSU2: Uniformity of salt distribution in the 20–40 cm soil layer.

By analysing the relationship between soil water-salt distribution characteristics and cotton seedling emergence rate (**Figure 10**), it was found that soil salinity in the 0–20 cm layer exhibited a significant negative correlation with cotton seedling emergence rate (p<0.05). Conversely, soil moisture content, moisture uniformity, salinity uniformity, and desalination rate in the 0–20 cm layer showed significant positive correlations with cotton seedling emergence rate (p<0.05). In the 20–40 cm soil layer, soil salinity, moisture content, moisture uniformity, salinity uniformity, and desalination rate showed no correlation with cotton emergence rate (p>0.05).

**Figure 11:**
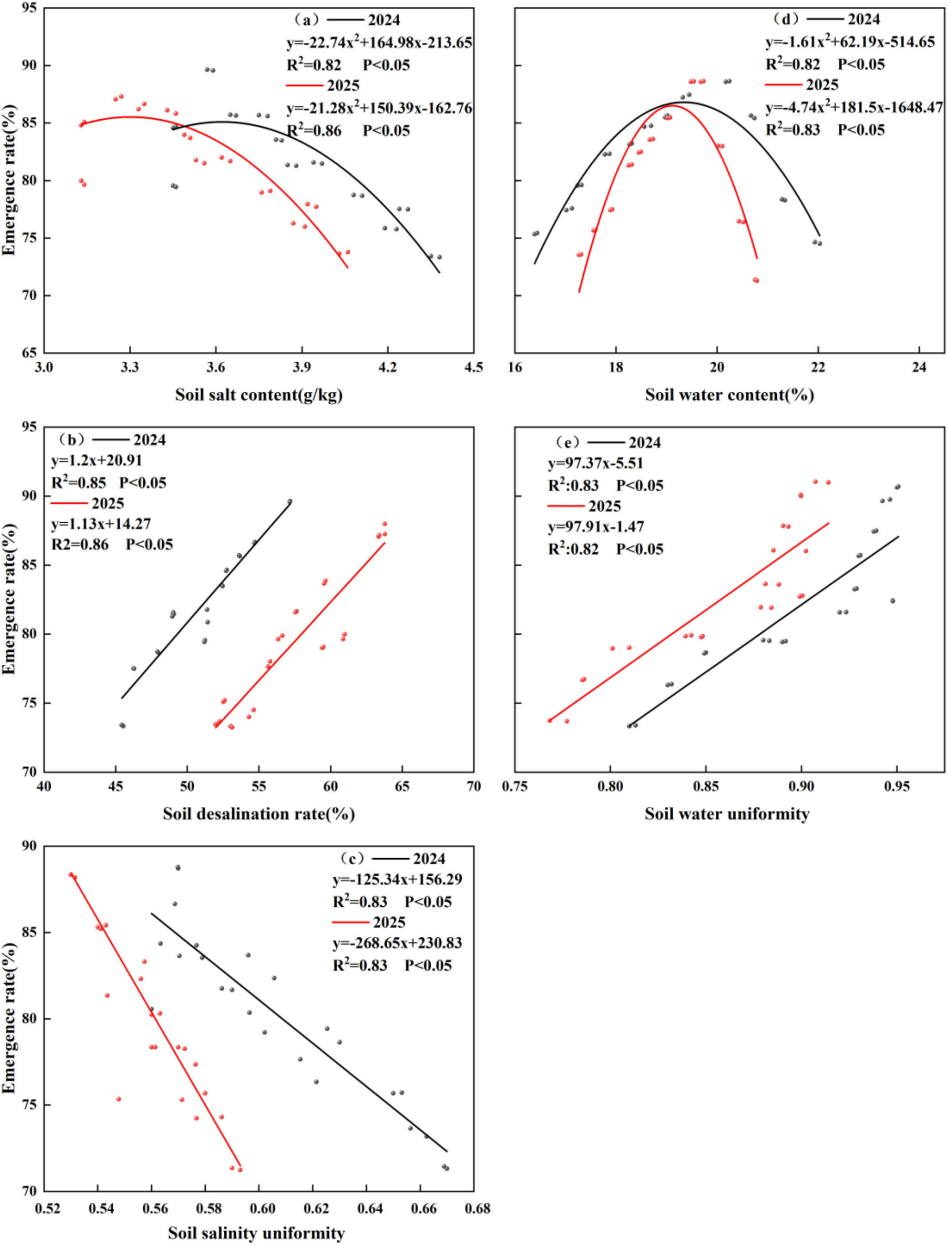
Regression analysis of soil water and salt characteristics at 0–20 cm depth in cotton fields and cotton seedling emergence rate. **(a–e)** respectively present the linear regression equations for soil salinity, soil desalination rate, soil salinity uniformity coefficient, soil moisture content, and soil moisture uniformity coefficient against cotton seedling emergence rate.

Further analysis revealed a significant non-linear relationship (R²>0.83) between soil salinity and moisture content in the 0–20 cm layer and cotton emergence rate (**Figure 11**). Emergence rate initially increased with rising soil salinity and moisture content before declining. The desalination efficiency and uniformity of water distribution in the 0–20 cm soil layer showed a significant positive linear correlation with cotton emergence rate (R²>0.82). Conversely, the uniformity of soil salinity distribution exhibited a significant negative linear correlation with cotton emergence rate (R²=0.83).

## 4 Discussion

This study found that rationally optimising irrigation volume and frequency can improve soil water-salt distribution characteristics, thereby enhancing seedling emergence in “dry sowing and wet emergence” cotton fields. Under the study conditions, the P2W2 treatment—combining increased irrigation frequency during the emergence stage with a 30% reduction in irrigation volume—effectively expanded the soil moisture coverage within the 0–20 cm layer. This enhanced soil water-salt uniformity, reducing soil salinity in the 0–20 cm layer by 3.41–11.85% compared to other treatments at 12 days post-sowing. The desalination effect increased by 20.17% to 35.94%, resulting in a emergence rate that was 5.79% to 13.91% higher than that of other treatments.

### 4.1 Effects of irrigation volume and frequency on water movement and distribution characteristics

Irrigation strategies are the dominant factors regulating soil water migration (Xu et al., 2017; An et al., 2025). Moderately increasing irrigation volume extends both vertical and horizontal water penetration distances, maintains sustained moisture in the surface soil layer, and expands the wetted area, thereby enhancing water distribution uniformity (Feng et al., 2023; Sampathkumar et al., 2012). Under the synergistic regulation of irrigation volume and frequency, a high-frequency, low-volume irrigation pattern helps retain more water in the upper root zone. Soil moisture content gradually decreases with increasing distance from the drip tape (Zalacáin et al., 2019). Higher irrigation volumes can slow the rate of soil moisture decline and improve water distribution uniformity (Wang et al., 2006; Qi et al., 2020). Under the conditions of this study, both irrigation rate and frequency during seedling emergence significantly influenced soil moisture migration and distribution patterns. Increasing irrigation frequency while moderately reducing irrigation rate effectively maintained soil moisture content in the 0–20 cm layer and enhanced uniformity of soil moisture distribution. This finding aligns with the conclusions of (Tian et al., 2022). Compared to other treatments, the P2W2 treatment increased soil moisture content by 2.86%–12.05% in the 0–20 cm soil layer and improved moisture uniformity by 3.14%–11.82%. Additionally, this study found that under “dry sowing and wet emergence” conditions, when irrigation volume reached 20 mm during seedling emergence, further increasing irrigation volume enhanced horizontal diffusion of the wetting front while reducing vertical infiltration range. Soil moisture content at the 0–30 cm horizontal depth increased by 6.37% in the P2W3 treatment group compared to the P2W2 group. The migration pattern of the wetting front during irrigation is jointly regulated by irrigation volume and initial soil conditions; increasing irrigation volume promotes its horizontal development (Han et al., 2025). The phenomenon observed in this study may stem from increased irrigation raising the soil matric potential, causing downward flow velocity to lag behind lateral transport rates. This disruption of the equilibrium between downward seepage and matric potential ultimately enhances the horizontal expansion of soil moisture content, a phenomenon consistent with both theoretical and observational findings on unsaturated flow in soil (Cote et al., 2003; Hu et al., 2025).

### 4.2 Effects of irrigation volume and frequency on salt migration and distribution characteristics

Irrigation volume determines the depth of salt leaching, while irrigation frequency influences the distribution of salts; together, they regulate the distribution characteristics of soil salinity (Du et al., 2023; Liu et al., 2025a). Under “dry sowing and wet emergence” conditions, lower irrigation volumes readily exacerbate surface salt accumulation due to increased surface evaporation, thereby intensifying soil salinization (Sun et al., 2019). Moderately increasing irrigation volume can extend the diffusion range of the wetting front, thereby promoting the leaching of surface salts and enhancing desalination in the topsoil (Guan et al., 2013). The frequency of irrigation plays a particularly crucial role in regulating salt redistribution patterns, as it fundamentally alters the dynamic equilibrium between water movement and evaporation processes (Chen et al., 2018). High-frequency irrigation maintains elevated soil matric potential in the root zone, not only directly promoting downward salt migration with water flow but more importantly, continuously suppressing surface salt accumulation caused by surface water evaporation. This effectively prevents salt accumulation in the seed germination layer or the primary root zone of crops (Wieber et al., 2025). In contrast, although low-frequency irrigation involves larger single-application volumes and can generate significant leaching effects in the short term post-irrigation, intense soil surface evaporation during extended irrigation intervals rapidly reverses the direction of salt movement. This process causes salts leached into deeper soil layers to migrate back upward with water, accumulating at the soil surface and ultimately forming salt crusts or salt patches that impede seedling emergence (Ding et al., 2024). Thus, irrigation frequency essentially regulates the cycle of vertical leaching- upwelling dynamics in the soil profile. Under this mechanism, high-frequency irrigation effectively enhances stable leaching of salts within the root zone by avoiding deep percolation caused by single excessive irrigation events (Gao et al., 2025). Under the conditions of this study, irrigation volume and frequency significantly influenced soil salinity migration and distribution characteristics. Increasing both irrigation volume and frequency effectively leached salts to the 30–40 cm soil layer, reduced soil salinity content and uniformity in the 0–20 cm layer, and enhanced desalination efficacy. Compared to other treatments, the P2W2 regimen enhanced soil salinity reduction in the 0–20 cm layer by 20.17%–35.34%, decreased salinity content by 3.78%–12.94%, and reduced salinity distribution uniformity by 3.49%–12.41%. Previous studies predominantly advocated that ‘high frequency + high irrigation volume’ yielded optimal desalination effects. However, this research observed during the cotton emergence stage in “dry sowing and wet emergence” fields that employing high-frequency irrigation combined with a 30% reduction in irrigation volume achieved 48.06% desalination in the 0–20 cm soil layer. representing a 6.78% increase over the ‘high frequency + high irrigation volume’ approach and exceeding other treatments by 20.17% to 35.34%. This discrepancy may stem from excessive irrigation in the W3 treatment causing partial water percolation into deeper layers, preventing adequate dissolution of salts within the 0–20 cm profile and hindering their migration to deeper zones. This reduced salt leaching efficiency in the 0–20 cm soil layer (Liu et al., 2025b). Conversely, the P2W2 combination achieved precise regulation of soil water and salts through ‘moderate irrigation volume + high frequency’, better meeting the requirements for controlling surface soil salinity during the seedling stage of cotton fields.

### 4.3 Effect of irrigation volume and frequency on cotton seedling emergence rate

The soil water-salt environment directly influences the emergence process of cotton seedlings, with an appropriate water-salt environment promoting seedling emergence (Tao et al., 2023). Under “dry sowing and wet emergence” conditions, irrigation volume and frequency are key factors regulating soil water-salt distribution patterns. Insufficient irrigation volume leads to inadequate salt leaching, exacerbating surface salt accumulation and elevating soil osmotic pressure. High-salinity stress not only hinders seed water uptake but, according to prior research, also disrupts normal seed metabolic initiation through ionic toxicity and osmotic stress, significantly reducing emergence rates (Terletskaya et al., 2023). Conversely, excessive irrigation leads to overly high soil moisture content, resulting in deteriorated root zone aeration. Soil hypoxia severely impairs seed aerobic respiration and energy supply, similarly reducing cotton seedling emergence rates (Lu et al., 2023). Through the synergistic effects of irrigation volume and frequency, this approach prevents both salt stress caused by water deficiency and soil degradation induced by waterlogging (Ertek et al., 2004). This pattern establishes an optimal water-salt environment in the root zone, creating favorable soil conditions for cotton seed germination and emergence, thereby significantly improving seedling emergence rates (Ma et al., 2017; Li et al., 2021). Through rational adjustment of irrigation volume and frequency, the P2W2 treatment demonstrated the following improvements compared to other treatments: soil moisture content in the 0–20 cm layer increased by 2.86%–12.05%, soil salinity decreased by 3.78%–12.94%, soil moisture distribution uniformity improved by 3.14%–11.82%, and increased salt leaching efficiency by 20.17% to 35.34%. Consequently, cotton emergence rates were 5.79% to 13.91% higher than in other treatments, this result is consistent with previous studies, which indicate that creating suitable and uniform water-salt conditions in the germination layer through irrigation management is a key physiological process for ensuring uniform crop emergence and improving emergence rates (Guang et al., 2019). Moreover, the emergence rate in 2024 was 1.84% higher than that in 2025 (**Figure 9**), which may be attributed to the poorer emergence conditions in 2025 due to frequent rainfall and lower temperatures during the germination period (Wang et al., 2025). Furthermore, this study found that beyond 20 mm of irrigation, continued increases in irrigation volume actually led to a declining trend in cotton seedling emergence rates. This phenomenon may stem from excessive soil moisture content when irrigation volume and frequency exceed specific thresholds, resulting in deteriorated soil aeration. This, in turn, triggers soil compaction that inhibits seed germination metabolism, ultimately reducing emergence rates (Garg et al., 2021). This study elucidates the effects of irrigation frequency and volume on soil water and salt distribution characteristics during the cotton emergence period under dry sowing and wet emergence conditions. Future research may focus on investigating the dynamic changes in physiological indicators of cotton under varying water-salinity conditions, further elucidating the physiological mechanisms underlying irrigation strategy regulation of seedling emergence. By integrating numerical modeling to simulate soil water-salinity transport processes under different irrigation strategies, this approach aims to optimize irrigation schemes for areas with varying degrees of salinization in southern Xinjiang, thereby providing scientific support for the sustainable development of the local cotton industry.

## 5 Conclusion

This study confirms that under “dry sowing and wet emergence” conditions, the P2W2 treatment (irrigating 15 mm on both the first and eighth days after sowing) demonstrated the best overall performance in coordinating water and salt distribution in the root zone and ensuring cotton seedling emergence, achieving a emergence rate of 90.63%. This treatment maintained soil moisture content at 18.36%–19.82% in the 0–20 cm soil layer while controlling soil salinity below 3.65 g/kg, thereby creating a balanced water-salt microenvironment conducive to seed germination and seedling emergence. This water-salt threshold (soil moisture ≥18.36%, salinity <3.65 g/kg) serves as a management indicator for achieving stable emergence (emergence rate >85%). These findings provide clear and reliable theoretical basis and technical guidance for optimizing the “dry sowing and wet emergence” irrigation system in arid, saline-affected cotton areas of southern Xinjiang, thereby promoting water conservation, seedling protection, stable production, and increased yields.

## Data Availability

The original contributions presented in the study are included in the article/supplementary material. Further inquiries can be directed to the corresponding author.

## References

[B1] An Z. Wang R. M. Chai M. T. Li X. Li W. C. (2025). Influence of spring and winter irrigation on salt transport in soil during freeze-thaw cycles. J. Hydrology 663, 134140. doi: 10.1016/j.jhydrol.2025.134140, PMID: 41727822

[B2] Cao Y. D. Zhang W. Ren J. Z. (2020). Efficiency analysis of the input for water-saving agriculture in China. Water 12, 207. doi: 10.3390/w12010207, PMID: 41725453

[B3] Chen W. L. Jin M. G. Ferré T. P. A. Liu Y. F. Xian Y. Shan T. R. . (2018). Spatial distribution of soil moisture, soil salinity, and root density beneath a cotton field under mulched drip irrigation with brackish and fresh water. Field Crops Res. 215,207–221. doi: 10.1016/j.fcr.2017.10.019, PMID: 41727822

[B4] Cote C. M. Bristow K. L. Charlesworth P. B. Cook F. J. Thorburn P. J. (2003). Analysis of soil wetting and solute transport in subsurface trickle irrigation. Irrigation Sci. 22, 143–156. doi: 10.1007/s00271-003-0080-8, PMID: 41728210

[B5] Dai J. L. Cui Z. P. Zhang Y. J. Zhan L. J. Nie J. J. Cui J. Q. . (2024). Enhancing stand establishment and yield formation of cotton with multiple drip irrigation during emergence in saline fields of Southern Xinjiang. Field Crops Res. 315, 109482. doi: 10.1016/j.fcr.2024.109482, PMID: 41727822

[B6] Ding Y. Ma J. Q. Zhang J. H. Bai Y. G. Cui B. F. Hao X. P. . (2024). Response of photosynthesis, population physiological indexes, and yield of cotton in dry areas to the new technology of “dry sowing and wet emergence. Front. Plant Sci. 15. doi: 10.3389/fpls.2024.1487832, PMID: 39483670 PMC11526576

[B7] Du Y. Q. Liu X. F. Zhang L. Zhou W. (2023). Drip irrigation in agricultural saline-alkali land controls soil salinity and improves crop yield: Evidence from a global meta-analysis. Sci. Total Environ. 880, 163226. doi: 10.1016/j.scitotenv.2023.163226, PMID: 37019232

[B8] Ertek A. Şensoy S. Küçükyumuk C. Gedik İ. (2004). Irrigation frequency and amount affect yield components of summer squash (Cucurbita pepo L.). Agric. Water Manage. 67, 63–76. doi: 10.1016/j.agwat.2003.12.004, PMID: 41727822

[B9] Feng L. Wan S. M. Zhang Y. L. Dong H. Z. (2024). Xinjiang cotton: Achieving super-high yield through efficient utilization of light, heat, water, and fertilizer by three generations of cultivation technology systems. Field Crops Res. 312, 109401. doi: 10.1016/j.fcr.2024.109401, PMID: 41727822

[B10] Feng X. Y. Pu J. X. Liu H. J. Wang D. Liu Y. H. Qiao S. T. . (2023). Effect of fertigation frequency on soil nitrogen distribution and tomato yield under alternate partial root-zone drip irrigation. J. Integr. Agric. 22, 897–907. doi: 10.1016/j.jia.2022.09.002, PMID: 41727822

[B11] Gao F. K. Han Q. S. Sun J. S. Zhao Q. Y. Yang G. Zhang X. B. . (2025). Side soil covering and high-frequency low-volume irrigation improve cotton seedling emergence by altering soil physical properties and salinity. Soil Tillage Res. 258, 107025. doi: 10.1016/j.still.2025.107025, PMID: 41727822

[B12] Garg A. Huang H. Cai W. L. Reddy N. G. Chen P. N. Han Y. F. . (2021). Influence of soil density on gas permeability and water retention in soils amended with in-house produced biochar. J. Rock Mechanics Geotechnical Eng. 13, 593–602. doi: 10.1016/j.jrmge.2020.10.007, PMID: 41727822

[B13] Green D. Pattison I. (2022). Christiansen uniformity revisited: Re-thinking uniformity assessment in rainfall simulator studies. Catena 217, 106424. doi: 10.1016/j.catena.2022.106424, PMID: 41727822

[B14] Guan H. J. Li J. S. Li Y. F. (2013). Effects of drip system uniformity and irrigation amount on water and salt distributions in soil under arid conditions. J. Integr. Agric. 12, 924–939. doi: 10.1016/s2095-3119(13)60310-x, PMID: 41717493

[B15] Guang J. F. Shao X. H. Miao Q. S. Yang X. Gao C. Ding F. Z. . (2019). Effects of irrigation amount and irrigation frequency on flue-cured tobacco evapotranspiration and water use efficiency based on three-year field drip-irrigated experiments. Agronomy 9, 624. doi: 10.3390/agronomy9100624, PMID: 41725453

[B16] Han X. D. Qian Y. Z. Zhu Y. Ye M. Zhao T. X. Song H. Y. (2025). Effects of autumn irrigation timing and amounts on soil water and salt component migration in seasonally frozen soils. Agric. Water Manage. 317, 109639. doi: 10.1016/j.agwat.2025.109639, PMID: 41727822

[B17] He Y. J. Li X. W. Jin M. G. (2023). Temporal and spatial assessment of soil salinity post-flood irrigation: A guide to optimal cotton sowing timing. Agronomy 13, 2246. doi: 10.3390/agronomy13092246, PMID: 41725453

[B18] He Z. J. Cao H. X. Hu Q. Y. Qi C. Li Z. J. (2025). Salt leaching with alternate surface and subsurface drip irrigation enhance cotton yield, water use efficiency, desalination rate, desalination efficiency and economic benefit. Field Crops Res. 325, 109804. doi: 10.1016/j.fcr.2025.109804, PMID: 41727822

[B19] Hu Q. Y. Cao H. X. He Z. J. Shi H. L. Ren Z. W. Qi C. (2025). Effects of leaching amounts and drip irrigation types on water-salt distribution and seed cotton yield in northern Xinjiang, China. Field Crops Res. 328, 109947. doi: 10.1016/j.fcr.2025.109947, PMID: 41727822

[B20] Hu Q. L. Yang Y. H. Han S. M. Wang J. S. (2019). Degradation of agricultural drainage water quantity and quality due to farmland expansion and water-saving operations in arid basins. Agric. Water Manage. 213, 185–192. doi: 10.1016/j.agwat.2018.10.019, PMID: 41727822

[B21] Isayenkov S. V. Maathuis F. J. M. (2019). Plant salinity stress: many unanswered questions remain. Front. Plant Sci. 10. doi: 10.3389/fpls.2019.00080, PMID: 30828339 PMC6384275

[B22] Jia F. F. Zeng D. Zhou B. Lv T. Li W. H. Chen T. . (2025). Water-fertilizer optimization significantly improved cotton yield under dry-sowing and wet-emergence pattern: The case study in arid area of Southern Xinjiang, China. Agric. Water Manage. 322, 109998. doi: 10.1016/j.agwat.2025.109998, PMID: 41727822

[B23] Jia Y. Yang B. F. Han Y. C. Wang G. P. Su T. L. Li X. F. . (2024). Enhanced cotton yield and fiber quality by optimizing irrigation amount and frequency in arid areas of northwest China. Agronomy 14, 266. doi: 10.3390/agronomy14020266, PMID: 41725453

[B24] Li D. F. (2020). Quantifying water use and groundwater recharge under flood irrigation in an arid oasis of northwestern China. Agric. Water Manage. 240, 106326. doi: 10.1016/j.agwat.2020.106326, PMID: 41727822

[B25] Li Y. Feng Q. Li D. Li M. Ning H. Han Q. . (2022). Water-Salt Thresholds of Cotton (Gossypium hirsutum L.) under Film Drip Irrigation in Arid Saline-Alkali Area. Agriculture 12. doi: 10.3390/agriculture1.2022.2111769, PMID: 41725453

[B26] Li N. N. Shi X. J. Zhang H. M. Shi F. Zhang H. X. Liang Q. . (2024). Optimizing irrigation strategies to improve the soil microenvironment and enhance cotton water productivity under deep drip irrigation. Agric. Water Manage. 305. doi: 10.1016/j.agwat.2024.109095, PMID: 41727822

[B27] Li S. Wu M. Jia Z. H. Luo W. Fei L. J. Li J. S. (2021). Influence of different controlled drainage strategies on the water and salt environment of ditch wetland: A model-based study. Soil Tillage Res. 208, 104894. doi: 10.1016/j.still.2020.104894, PMID: 41727822

[B28] Liang J. P. Shi W. J. (2021). Cotton/halophytes intercropping decreases salt accumulation and improves soil physicochemical properties and crop productivity in saline-alkali soils under mulched drip irrigation: A three-year field experiment. Field Crops Res. 262, 108027. doi: 10.1016/j.fcr.2020.108027, PMID: 41727822

[B29] Liang Y. K. Abulaiti M. Zhang L. Jing L. K. Gao Y. Huang W. X. . (2025). Drip-applied rhamnolipid ameliorates soil physicochemical properties and enhances cotton yield under dry sowing with wet emergence cultivation. Ind. Crops Products 233, 121463. doi: 10.1016/j.indcrop.2025.121463, PMID: 41727822

[B30] Liu J. W. Sun L. Zhu X. J. Deng H. X. Fan Y. M. Huang Q. Z. . (2025a). Effects of different annual irrigation strategies on soil water, salt, nitrogen leaching, and sunflower growth in saline soils of arid regions. Agric. Water Manage. 318, 109692. doi: 10.1016/j.agwat.2025.109692, PMID: 41727822

[B31] Liu X. Q. Lv F. Pei S. Z. Mu Y. F. Fan J. L. He S. . (2025b). Effects of irrigation management on soil water-heat-salinity dynamics and water productivity of winter jujube in saline-alkaline soils under a plastic-shed system. Agric. Water Manage. 321. doi: 10.1016/j.agwat.2025.109907, PMID: 41727822

[B32] Lu S. F. Han Z. J. Xu L. Lan T. G. Wei X. Zhao T. Y. (2023). On measuring methods and influencing factors of air permeability of soils: An overview and a preliminary database. Geoderma 435, 116509. doi: 10.1016/j.geoderma.2023.116509, PMID: 41727822

[B33] Ma J. Q. Ding Y. Zhang J. H. Bai Y. G. Cui B. F. Hao X. P. . (2024). Impact of “Dry sowing and wet emergence” Water regulation on physiological growth characteristics and water productivity of cotton fields in southern Xinjiang province. Agronomy 14, 734. doi: 10.3390/agronomy14040734, PMID: 41725453

[B34] Ma J. Q. Ding Y. Zhang J. H. Fan K. Bai Y. G. Cui B. F. . (2025). Exploring the response relationship between crop rooting, seedling emergence and soil water, heat and salt environmental factors in dry sowing wet emergent cotton fields. Ind. Crops Products 231, 121201. doi: 10.1016/j.indcrop.2025.121201, PMID: 41727822

[B35] Ma L. Ahuja L. R. Islam A. Trout T. J. Saseendran S. A. Malone R. W. (2017). Modeling yield and biomass responses of maize cultivars to climate change under full and deficit irrigation. Agric. Water Manage. 180, 88–98. doi: 10.1016/j.agwat.2016.11.007, PMID: 41727822

[B36] Nabi G. Mullins C. E. Montemayor M. B. Akhtar M. S. (2001). Germination and emergence of irrigated cotton in Pakistan in relation to sowing depth and physical properties of the seedbed. Soil Tillage Res. 59, 33–44. doi: 10.1016/S0167-1987(00)00182-3, PMID: 41334505

[B37] Qi D. L. Hu T. T. Song X. (2020). Effects of nitrogen application rates and irrigation regimes on grain yield and water use efficiency of maize under alternate partial root-zone irrigation. J. Integr. Agric. 19, 2792–2806. doi: 10.1016/s2095-3119(20)63205-1

[B38] Sampathkumar T. Pandian B. J. Mahimairaja S. (2012). Soil moisture distribution and root characters as influenced by deficit irrigation through drip system in cotton–maize cropping sequence. Agric. Water Manage. 103, 43–53. doi: 10.1016/j.agwat.2011.10.016, PMID: 41727822

[B39] Su F. M. Wu J. H. Wang D. Zhao H. H. Wang Y. H. He X. D. (2022). Moisture movement, soil salt migration, and nitrogen transformation under different irrigation conditions: Field experimental research. Chemosphere 300, 134569. doi: 10.1016/j.chemosphere.2022.134569, PMID: 35421440

[B40] Sun J. N. Yang R. Y. Zhu J. J. Pan Y. H. Yang M. Zhang Z. H. (2019). Can the increase of irrigation frequency improve the rate of water and salt migration in biochar-amended saline soil? J. Soils Sediments 19, 4021–4030. doi: 10.1007/s11368-019-02357-9, PMID: 41728210

[B41] Tao Q. B. Chen D. L. Bai M. J. Zhang Y. Q. Zhang R. Z. Chen X. F. . (2023). Hydrotime Model Parameters Estimate Seed Vigor and Predict Seedling Emergence Performance of Astragalus sinicus under Various Environmental Conditions. Plants (Basel) 12, 1876. doi: 10.3390/plants12091876, PMID: 37176935 PMC10180758

[B42] Terletskaya N. V. Erbay M. Zorbekova A. N. Prokofieva M. Y. Saidova L. T. Mamirova A. (2023). Influence of osmotic, salt, and combined stress on morphophysiological parameters of chenopodium quinoa photosynthetic organs. Agriculture 13, 1. doi: 10.3390/agriculture13010001, PMID: 41725453

[B43] Tian H. W. Bo L. Y. Mao X. M. Liu X. Y. Wang Y. Hu Q. Y. (2022). Modelling soil water, salt and heat dynamics under partially mulched conditions with drip irrigation, using HYDRUS-2D. Water 14, 2791. doi: 10.3390/w14182791, PMID: 41725453

[B44] Wang F. X. Kang Y. Liu S. P. (2006). Effects of drip irrigation frequency on soil wetting pattern and potato growth in North China Plain. Agric. Water Manage. 79, 248–264. doi: 10.1016/j.agwat.2005.02.016, PMID: 41727822

[B45] Wang S. Wang Z. H. Bai Y. G. Zhang J. H. Fang Z. (2025). Optimizing root zone environment to enhance technology stability of “dry sowing and wet emergence” in cotton fields of southern Xinjiang, China. Agric. Water Manage. 321, 109985. doi: 10.1016/j.agwat.2025.109895, PMID: 41727822

[B46] Wei K. Zhang J. H. Wang Q. J. Guo Y. Mu W. Y. (2022). Irrigation with ionized brackish water affects cotton yield and water use efficiency. Ind. Crops Products 175, 114244. doi: 10.1016/j.indcrop.2021.114244, PMID: 41727822

[B47] Wieber E. Kelly B. Nakabuye H. N. Bordovsky J. P. Ritchie G. L. Zhang F. Y. . (2025). Irrigation rate, timing, and cultivar effects on cotton yield and fiber quality. Agric. Water Manage. 323, 110029. doi: 10.1016/j.agwat.2025.110029, PMID: 41727822

[B48] Xiao C. Ji Q. Y. Chen J. Q. Zhang F. C. Li Y. Fan J. L. . (2023). Prediction of soil salinity parameters using machine learning models in an arid region of northwest China. Comput. Electron. Agric. 204, 107512. doi: 10.1016/j.compag.2022.107512, PMID: 41727822

[B49] Xu B. L. Shao D. G. Tan X. Z. Yang X. Gu W. Q. Li H. X. (2017). Evaluation of soil water percolation under different irrigation practices, antecedent moisture and groundwater depths in paddy fields. Agric. Water Manage. 192, 149–158. doi: 10.1016/j.agwat.2017.06.002, PMID: 41727822

[B50] Yu Q. L. Kang S. Z. Wu H. Song J. Wang H. Parsons D. (2025). Optimizing regional irrigation management in arid saline areas using a process-based hydro-salt-crop model and fallow strategy. Agric. Water Manage. 320, 109832. doi: 10.1016/j.agwat.2025.109832, PMID: 41727822

[B51] Zalacáin D. Martínez-Pérez S. Bienes R. García-Díaz A. Sastre-Merlín A. (2019). Salt accumulation in soils and plants under reclaimed water irrigation in urban parks of Madrid (Spain). Agric. Water Manage. 213, 468–476. doi: 10.1016/j.agwat.2018.10.031, PMID: 41727822

